# Advances in Scalp Microbiome Research: Molecular Insights into the Metabolism-Inflammation-Barrier Axis and Dandruff Pathogenesis

**DOI:** 10.3390/molecules31122093

**Published:** 2026-06-14

**Authors:** Le Deng, Xiao Ling, Li Li, Youjie He, Miaomiao Guo

**Affiliations:** 1 Beijing Key Laboratory of Plant Resources Research and Development, College of Light Industry Science and Engineering, Beijing Technology and Business University, Beijing 102488, China; 2 Beijing Lan Divine Technology Co, Ltd., Beijing 102200, China; 3 College of Biomass Science and Engineering, Sichuan University, Chengdu 610065, China

**Keywords:** dandruff, scalp microbiota, *Malassezia*, lipid metabolism, AhR signaling, ecological homeostasis

## Abstract

Dandruff (DF) is a prevalent, recurrent inflammatory scalp disorder increasingly recognized as a complex state of functional dysbiosis rather than a simple *Malassezia* overcolonization. The scalp microbiome is predominantly shaped by *Malassezia* species (*M. restricta* and *M. globosa*), *Cutibacterium*, and *Staphylococcus species*. Recent multi-omics evidence indicates that DF pathogenesis is driven by the destabilization of microbial interaction networks and strain-level functional heterogeneity, characterized by the disruption of the *C. acnes*/*S. epidermidis* balance and the opportunistic expansion of *Staphylococcus aureus*. Mechanistically, *Malassezia* utilizes its lipolytic repertoire to hydrolyze host sebum into irritant free fatty acids and peroxides. Concurrently, oxidative metabolites like squalene peroxide (SQOOH) penetrate the stratum corneum to activate the NF-κB and aryl hydrocarbon receptor (AhR) pathways, triggering a pro-inflammatory cascade that overexpresses keratins (K6/16/17) and downregulates filaggrin. This molecular cascade drives abnormal keratinocyte turnover and lipidomic remodeling, establishing a self-perpetuating “metabolism–inflammation–barrier disruption” pathological cycle. This review systematically elucidates the molecular etiology of DF as an ecological disorder driven by a tripartite imbalance among the microbiome, host physiology, and the environmental niche. We propose that next-generation therapeutic paradigms must transcend traditional antifungal eradication, focusing instead on targeted molecular intervention and microecological restoration to recalibrate overall scalp homeostasis.

## 1. Introduction

Dandruff (DF) is a common scalp condition characterized by excessive flaking of the stratum corneum, often accompanied by pruritus of varying severity. Clinically, DF presents as loosely adherent oily or dry scales on the scalp surface [1]. These manifestations reflect abnormal desquamation of corneocytes and are frequently associated with alterations in epidermal turnover compared with healthy scalp skin [2]. DF is generally considered part of the clinical spectrum of seborrheic dermatitis (SD). While both conditions share similar pathological features, DF is typically confined to the scalp with minimal visible inflammation, whereas SD may involve additional sebaceous areas and presents with more pronounced erythema and inflammatory responses [3].

Epidemiological studies indicate that dandruff affects a substantial proportion of the global population, with lifetime prevalence approaching 50% in adults [4]. Although DF is often regarded as a mild dermatological condition, it can cause persistent discomfort and cosmetic concerns, thereby negatively affecting quality of life and psychosocial well-being.

Accumulating evidence suggests that DF is closely associated with alterations in the scalp microbiome. Lipophilic yeasts of the genus *Malassezia*, particularly *Malassezia resrricta* and *Malassezia globosa* (*M. restricta* and *M. globosa*), are consistently identified as dominant fungal taxa and have long been implicated in dandruff pathophysiology [5]. However, recent microbiome-based studies indicate that dandruff is not solely driven by a single microorganism, but rather involves broader shifts in microbial community structure. In particular, changes in bacterial composition—especially in the relative abundance of *Staphylococcus* and *Cutibacterium*—have been reported to correlate with disease development [6].

Despite extensive research, the precise pathogenic mechanisms underlying DF remain incompletely understood. Current evidence suggests that multiple interacting factors contribute to disease development, including microbial lipid metabolism, the generation of irritant metabolites such as oleic acid, disruption of epidermal barrier integrity, and activation of local immune responses [7,8,9,10,11]. Notably, many of these mechanisms have been primarily characterized in experimental systems or SD cohorts, and their specific roles in dandruff require further clarification.

Current studies consistently identify three dominant microbial genera—*Malassezia*, *Staphylococcus*, and *Cutibacterium*—as key components of the scalp ecosystem [1,7,12,13]. However, most existing studies remain largely descriptive, focusing on differences in microbial abundance rather than functional interactions. The mechanistic links among microbial dysbiosis, host barrier function, and immune responses remain insufficiently defined [7,14,15].

Therefore, this review aims to systematically summarize recent advances in the understanding of dandruff from a microbiome-centered perspective, with a particular focus on the interactions among microbial communities, host factors, and the scalp microenvironment. In addition, emerging microbiome-based therapeutic strategies are discussed to provide insights into future directions for dandruff management.

Therefore, based on a comprehensive search of literature focused primarily on the past decade (2016–2026) indexed in PubMed and Web of Science, this paper systematically summarizes recent advances in dandruff research from a microbiome-centered perspective. We focus on the tripartite interactions among microbial communities, host factors, and the scalp microenvironment, while discussing emerging microbiome-targeted therapies to guide future precision dandruff management.

## 2. DF and Scalp Microbiota

### 2.1. Scalp Microecology

**Figure 1:**
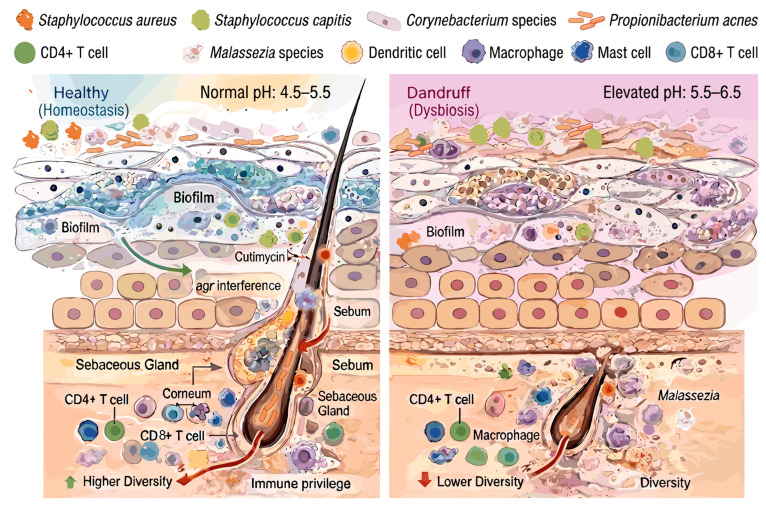
The Scalp Microecology and the Complex Interaction Niche. The schematic illustrates the specialized ecological niche of the scalp, characterized by an acidic pH mantle and abundant sebum secretion. (Top) Commensal bacteria (*Cutibacterium acnes*, *Staphylococcus* spp.) and fungi (*Malassezia* spp.) colonize the stratum corneum and the follicular infundibulum. (Interactions) Key ecological defenses are highlighted, including *C. acnes*-derived cutimycin and *S. capitis* interference with *S. aureus* quorum sensing. (Biofilm) Microbial persistence is enhanced by biofilm structures within the follicular interface. (Metabolism) Microbial lipases hydrolyze sebum into free fatty acids (FFAs), modulating the local immune response and maintaining the acidic microenvironment. Dandruff-associated dysbiosis is marked by a quantitative shift toward *Malassezia restricta* and a reduction in overall microbial diversity.

The scalp microbiome constitutes an integral component of the human cutaneous ecosystem, comprising diverse microorganisms—including bacteria, fungi, viruses, and mites—collectively referred to as the skin microbiota [7,16,17]. Compared with other skin sites, the scalp exhibits distinctive physiologic features, including abundant sebum secretion, relatively high humidity, mildly acidic pH, and a complex follicular architecture. These characteristics together create a specialized ecological niche that supports microbial colonization and shapes a characteristic scalp microbial community [17,18,19] (Figure 1).

Among these factors, sebum and the acidic microenvironment play central roles by providing metabolic substrates and favorable growth conditions for lipophilic microorganisms [17,18]. Species such as *Cutibacterium acnes* and *Malassezia* produce lipases that hydrolyze sebum triglycerides into free fatty acids, which not only support microbial growth but also contribute to maintaining the acidic skin surface environment [20,21]. This environment may further limit colonization by non-adapted microbial taxa.

The lipid-rich conditions of the scalp, together with its follicular structure, favor the proliferation of lipophilic microorganisms such as *Malassezia*, *Cutibacterium*, and *Corynebacterium*, which utilize sebum-derived lipids as energy sources [18]. The follicular infundibulum serves as a key interface for interactions between microorganisms and host keratinocytes [22].

Within this niche, microbial interactions contribute to community regulation; for example, certain *C. acnes* strains produce cutimycin, which inhibits *Staphylococcus aureus* and influences microbial competition [23].

Commensal microorganisms also contribute to maintaining microbial balance and host defense. Certain coagulase-negative staphylococci produce antimicrobial compounds that inhibit opportunistic pathogens such as *S. aureus* [24]. Within the scalp microenvironment, interactions among microorganisms play an important role in maintaining community stability. For example, certain commensal species such as *Staphylococcus capitis* can antagonize *Staphylococcus aureus* by interfering with the accessory gene regulator (agr) quorum-sensing system, thereby limiting pathogen colonization [25,26]. These findings highlight the role of commensal microbes in ecological defense.

In addition, biofilm formation by microorganisms such as *Staphylococcus epidermidis* and *Cutibacterium acnes* may enhance microbial adhesion and persistence within the scalp environment, contributing to their long-term colonization [27]. Microbial metabolites further mediate host–microbe interactions. Lipid-derived products and short-chain fatty acids produced by species such as *C. acnes* can influence keratinocyte function and modulate local immune responses [28,29,30,31,32,33]. These metabolites may also regulate microbial competition and contribute to maintaining the balance of the scalp microbiome [34,35].

Collectively, these microbial interactions—including competition, metabolic activity, and niche adaptation—are essential for maintaining scalp homeostasis, whereas disruption of these processes may contribute to microbial dysbiosis associated with dandruff.

### 2.2. Characteristics of Scalp Fungi and Their Associations with DF

Compared with other cutaneous sites, the scalp microbiota exhibits relatively low diversity, with the fungal community showing a highly skewed distribution dominated by yeasts of the genus *Malassezia* [15]. These fungi are present in both healthy individuals and patients with dandruff (DF) or seborrheic dermatitis (SD), although their relative abundance and overall fungal load are generally higher in affected individuals [36,37,38]. In healthy scalps, *Malassezia* may account for a substantial proportion of the microbial community, and this proportion often increases further under disease conditions.

Among *Malassezia* species, *M. restricta* and *M. globosa* are consistently identified as the predominant taxa on the human scalp, representing the majority of fungal sequences detected in molecular studies [39]. Other species, including *M. sympodialis*, *M. slooffiae*, and *M. dermatis*, are also detectable but typically occur at lower abundance [39].

The composition of the scalp mycobiome shows considerable inter-individual variability, influenced by host-related factors such as age, sex, ethnicity, and disease status [15,16,17,40,41,42], as well as environmental factors including climate and pollution [42].

Accumulating evidence suggests that *Malassezia* abundance is positively associated with dandruff. Clinical observations indicate that antifungal treatments targeting *Malassezia* can effectively alleviate symptoms, supporting its role in disease-associated dysbiosis [6]. Several studies have reported increased fungal burden in DF patients compared with healthy controls, although the relative proportions of individual species may vary [43]. Notably, *M. restricta* appears to be consistently dominant in both healthy and dandruff-affected scalps, but is often relatively enriched in DF-associated communities compared with other species such as *M. globosa* and *M. furfur* [43,44,45].

Some studies further suggest that fungal load may correlate with dandruff severity, although this relationship remains influenced by methodological differences and clinical heterogeneity [39,46,47]. Overall, current evidence indicates that dandruff is not simply associated with the presence of *Malassezia*, but rather with quantitative and compositional shifts within the fungal community, particularly involving *M. restricta*. However, the precise relationship between fungal abundance and disease severity remains to be fully clarified. Recent paradigms in skin microecology suggest that these host chronological and pathological transitions fundamentally reconstruct the local biophysical niche, driving sharp functional variations across different microbial configurations [18,48].

### 2.3. Characteristics of Scalp Bacteria and Their Associations with DF

Bacterial diversity on the scalp is relatively low compared with other skin sites, with microbial populations typically present at moderate densities of approximately 10^4^–10^5^ cells/cm^2^ [6,16]. The scalp bacterial community is predominantly composed of *Cutibacterium* (formerly *Propionibacterium*), particularly *Cutibacterium acnes*, followed by *Staphylococcus* species such as *Staphylococcus epidermidis* [7,17]. In addition, lower-abundance genera—including *Corynebacterium*, *Streptococcus*, *Acinetobacter*, *Prevotella*, *Pseudomonas*, and *Lawsonella*—also contribute to the overall microbial composition [17,18].

Although dandruff (DF) has traditionally been linked to *Malassezia*, increasing evidence suggests that bacterial communities also play an important role in scalp dysbiosis. Studies across different populations consistently report that *Cutibacterium* and *Staphylococcus* remain dominant regardless of disease status [7,17]. However, alterations in bacterial community structure—rather than absolute abundance—appear to be associated with DF, and in some cases show stronger correlations with disease severity than fungal changes [7,14]. In particular, increased abundance of *Staphylococcus epidermidis* has been reported in DF patients, indicating a potential role in disease-associated microbial shifts [7,14,17]. Crucially, recent multi-omics insights indicate that these resident taxa must be evaluated at the strain level rather than the species level, as distinct strains within the same species harbor high genomic and phenotypic heterogeneity [48]. For instance, while commensal strains of *C. acnes* and *S. epidermidis* generally maintain barrier homeostasis, specific pro-inflammatory strains can shift toward opportunistic pathogenicity under altered host physiological conditions (such as immunosenescence or lipid dysregulation), actively secreting tissue-destructive enzymes like proteases and elastases that degrade the host’s extracellular matrix (ECM) and lower the threshold for clinical inflammation [48,49,50].

Interactions between bacterial species contribute to maintaining microbial equilibrium. For example, *Cutibacterium* can inhibit *Staphylococcus* through bacteriocin production, whereas *Staphylococcus* may suppress *Cutibacterium* via metabolic activities such as glycerol fermentation [51,52]. These reciprocal interactions are important for maintaining ecological balance on the scalp.

In dandruff conditions, this balance appears to be disrupted. Several studies have reported shifts in bacterial community structure in affected regions, including altered *Staphylococcus* composition, with decreases in *S. epidermidis* and increases in *Staphylococcus aureus* in some cases [53,54]. This breakdown often mirrors a host-microbiome mismatch where host barrier failure and altered sebum chemistry accelerate the decline of beneficial symbionts while promoting the overgrowth of opportunistic, keratolytic taxa like *Corynebacterium* species, converting a mutualistic niche into a dense, inflammatory entanglement [48,55]. Such alterations may extend beyond visibly affected areas, suggesting broader ecosystem-level changes.

Overall, current evidence indicates that dandruff is associated with bacterial dysbiosis characterized by shifts in community structure and interspecies interactions, rather than simple changes in bacterial abundance. However, the precise role of bacteria in DF pathogenesis remains to be fully elucidated.

### 2.4. Association Between Scalp Microbiota and DF

Dandruff (DF) is increasingly recognized as a disorder involving complex interactions within the scalp microbial ecosystem rather than the overgrowth of a single microorganism. Disruptions in microbial co-occurrence and mutual exclusion networks have been associated with scalp dysbiosis in DF and seborrheic dermatitis (SD) [17]. The relationships between representative scalp microorganisms and DF reported in previous studies are summarized in Table 1.

For example, synergistic interactions between certain bacterial and fungal species, such as *Bacillus* sp. and non-pathogenic *Malassezia*, may contribute to maintaining microbial homeostasis [58].

Network-based analyses further support the importance of microbial interactions. Co-occurrence network studies have revealed dense and highly interconnected relationships among scalp microorganisms, with reduced network stability observed in DF patients compared with healthy individuals [45]. In particular, *M. restricta* shows antagonistic associations with other *Malassezia* species and several bacterial genera, while beneficial taxa such as *Lactobacillus* display weakened inhibitory interactions with dominant microbes in DF. These findings suggest that disruption of microbial interaction networks, rather than simple compositional changes, may underlie DF-associated dysbiosis.

Within the follicular microenvironment, *Cutibacterium acnes* appears to play a key role in maintaining microbial balance. Its metabolite, propionic acid, can inhibit the growth of *M. restricta* and *Staphylococcus epidermidis*, potentially through iron-limiting effects [59]. However, under DF conditions, factors such as barrier disruption and oxidative stress may impair *C. acnes* homeostasis, thereby facilitating *Malassezia* overgrowth. These observations highlight the importance of considering the scalp and hair follicle as an integrated ecological unit.

Core microbiome analyses indicate that healthy scalps are typically dominated by *C. acnes* and *S. epidermidis*, with fungal communities mainly composed of *M. restricta* and *M. globosa*. In contrast, DF is often associated with increased abundance of *Malassezia* (particularly *M. restricta*) and *Staphylococcus*, along with reduced levels of *Cutibacterium* [14,47]. Some studies further suggest that *M. globosa* may show negative associations with disease severity, indicating potential functional divergence among *Malassezia* species.

Functional metagenomic analyses reveal additional differences between healthy and DF-associated microbiomes. DF-related fungal communities show enrichment in host interaction pathways such as N-glycan biosynthesis, largely attributed to *M. restricta*, whereas healthy microbiota are enriched in amino acid and fatty acid metabolism. At the bacterial level, pathways related to vitamin metabolism, including biotin and vitamin B6 biosynthesis, are more prominent in healthy communities, potentially linked to *Cutibacterium* species [14,47]. To put this into perspective, the functional differences between healthy and dandruff-associated microbiomes are stark: in healthy communities, microbial functional pathways are heavily enriched in biosynthetic and maintenance housekeeping operations, including amino acid metabolism, fatty acid elongation, and essential vitamin synthesis pathways, which support host keratinocyte homeostasis. Conversely, in dandruff-associated communities, metabolic pathways shift aggressively toward host colonization and survival, characterized by a distinct enrichment in host interaction pathways such as N-glycan biosynthesis (primarily driven by *M. restricta*), glycerolipid metabolism, and virulent extracellular enzyme export pathways, facilitating nutrient harvesting at the cost of the host tissue. This biochemical divergence underscores the profound role of microbial-derived metabolites and postbiotic signaling in regulating host pathology [48,60]. Specifically, this regulatory network operates via a dual paradigm: under healthy homeostasis, commensal-derived metabolites (such as propionic acid and short-chain fatty acids [SCFAs] secreted by *Cutibacterium acnes*, and lipoteichoic acids) act as "biological shields" that neutralize reactive oxygen species (ROS), suppress host tissue-destructive matrix metalloproteinases (MMPs), and actively reinforce lamellar lipid synthesis to lock in barrier macromolecular integrity [48,61]. In sharp contrast, during dandruff-associated dysbiosis, the depletion of these protective postbiotic signals, combined with *Malassezia*-mediated hydrolysis of host sebum, leads to an influx of irritating fungal free fatty acids (FFAs). This pathological influx induces severe intracellular oxidative stress and mitochondrial distress in keratinocytes, triggering inflammatory downstream cascades and causing advanced macromolecular barrier collapse [48,61].

Several studies have also reported that bacterial α-diversity on the scalp may be higher in patients with DF or SD compared with healthy individuals, with phyla such as Actinobacteria and Firmicutes commonly dominating these communities. In many cohorts, increased relative abundance of *Staphylococcus* and *Streptococcus* and reduced abundance of *Cutibacterium* have been observed in affected skin regions, suggesting the presence of bacterial dysbiosis associated with barrier dysfunction [38].

Microbial interactions also influence scalp barrier function. Increased abundance of *Staphylococcus* has been associated with barrier impairment and elevated transepidermal water loss (TEWL) [24]. Meanwhile *Malassezia* species may modulate *S. aureus* colonization through changes in local pH and enzyme secretion [62,63,64,65]. Specifically, *M. globosa* has been reported to secrete aspartic proteases capable of disrupting *S. aureus* biofilm formation [66].

In contrast, antagonistic interactions between *Cutibacterium* and *Staphylococcus*—mediated by bacteriocins and metabolic competition—may contribute to microbial balance [17,51,52]. Notably, co-colonization by *M. restricta* and *C. acnes* has been reported to cause less barrier disruption than *M. restricta* alone, and *C. acnes* abundance has been positively associated with scalp hydration [17,63].

Overall, current evidence suggests that DF arises from disruptions in microbial interaction networks and host–microbe relationships rather than the activity of a single pathogen. From this ecological perspective, strategies aimed at restoring microbial balance and reinforcing host–microbiota interactions may represent promising therapeutic approaches.

## 3. Molecular Etiology and Pathogenesis of Dandruff

### 3.1. Theoretical Framework: From Clinical Manifestation to Molecular Dyshomeostasis

Dandruff (DF) is a chronic, recurrent inflammatory scalp disorder characterized by abnormal desquamation of the stratum corneum and pruritus. Beyond physical discomfort, it frequently causes significant psycho-social distress. Clinically, DF presents as white or yellowish scales adhering to the scalp, occasionally extending to the nasolabial folds and retroauricular regions [67]. While DF shares pathological features with Seborrheic Dermatitis (SD) and responds to similar treatments, they differ in severity and distribution: DF is primarily non-inflammatory scaling confined to the scalp, whereas SD involves broader anatomical regions—such as the face and upper chest—accompanied by visible erythema and pronounced inflammation [68].

Current research suggests DF arises from the interplay of three primary factors:

1. Microbial Dysbiosis: While *Malassezia* colonization is a hallmark, recent evidence highlights the role of bacterial community restructuring (e.g., shifts in *Staphylococcus* and *Cutibacterium* abundance) [1].
2. Sebum Dysregulation: Alterations in sebum quantity and composition, particularly free fatty acid (FFA) profiles, fuel microbial metabolic activity.
3. Host susceptibility: Genetic variations affecting immune responses and epidermal barrier integrity determine individual sensitivity to microbial stimuli [69].

However, defining the precise causal architecture within this triad—particularly whether microbial components like *Malassezia* act as primary causal agents or secondary associative bystanders—requires critical evaluation. Historically, a strict causal role was inferred from the therapeutic success of topical antifungals [1]. Yet, from a pharmacological standpoint, therapeutic resolution does not automatically equate to etiological initiation. Successfully suppressing fungal load merely proves that *Malassezia* acts as an obligatory amplifier of the ongoing desquamative process; it fails to demonstrate that the yeast is the upstream initiator that originally disrupted host homeostasis.

Similarly, experimental models demonstrating that the exogenous application of *Malassezia* metabolites (e.g., oleic acid) replicates DF-like flaking in vivo confirm a potent pathogenic potential [67,70]. However, these high-concentration, acute chemical provocations reflect artificial induction rather than the gradual, endogenous shift in a resident commensal community, leaving a chronological gap in our understanding of spontaneous pathogenesis.

Recent advances in scalp microbiome research have further expanded the understanding of DF pathogenesis, shifting the focus from isolated pathogens to collective network shifts. Increasing evidence indicates that bacterial community restructuring, such as alterations in the relative abundance of *Cutibacterium* and *Staphylococcus*, is closely associated with clinical manifestations [17]. This multi-microbial involvement suggests that assigning exclusive, deterministic causality to a single fungal genus may be a historical over-simplification heavily indexed on culture-dependent observations.

Consequently, rather than being attributed solely to *Malassezia* overgrowth—which paradoxically occurs in healthy individuals without inducing clinical symptoms—DF is now understood as a systemic failure of the scalp microenvironment involving an integrated, non-linear framework of microbes, host immunity, and barrier function. Within this framework, *Malassezia* cannot be neatly categorized either as a singular upstream cause or a redundant downstream associate; instead, it operates as a context-dependent pathogen whose active virulence is strictly gated by host susceptibility and concurrent bacterial dysbiosis. Crucially, dandruff severity is driven by a non-linear positive feedback loop encompassing three interconnected dimensions (Microbiome-ROS-Barrier): (1) Microbiome Alteration to Barrier Damage: Driven by altered host physiological or lipid states, the overgrowth of specific pro-inflammatory bacterial strains and *Malassezia* leads to the hyper-secretion of tissue-destructive enzymes (proteases, lipases, and elastases) that actively degrade the extracellular matrix (ECM) and structural proteins, compromising physical barrier integrity. (2) Barrier Failure to Oxidative Stress: The compromised barrier increases transepidermal water loss (TEWL) and permits xenobiotics/irritating FFAs to penetrate deeper epidermal layers, generating massive intracellular ROS accumulations and mitochondrial distress. (3) Oxidative Stress back to Dysbiosis: The resulting microenvironmental oxidative stress and localized cell death destroy the niche for beneficial symbionts, further promoting the colonization of opportunistic, keratolytic taxa (e.g., *Corynebacterium*), which self-perpetuates the disease and increases clinical flaking and inflammation severity".

### 3.2. Role of Malassezia in DF Formation

Colonization of the scalp by *Malassezia* species has long been considered an important factor associated with the development of dandruff (DF), a view supported by the clinical efficacy of antifungal treatments in alleviating DF symptoms [1]. As a dominant member of the cutaneous microbiota, *Malassezia* transitions from a commensal to a pathogen when the scalp microenvironment is compromised [16,39].

**Figure 2:**
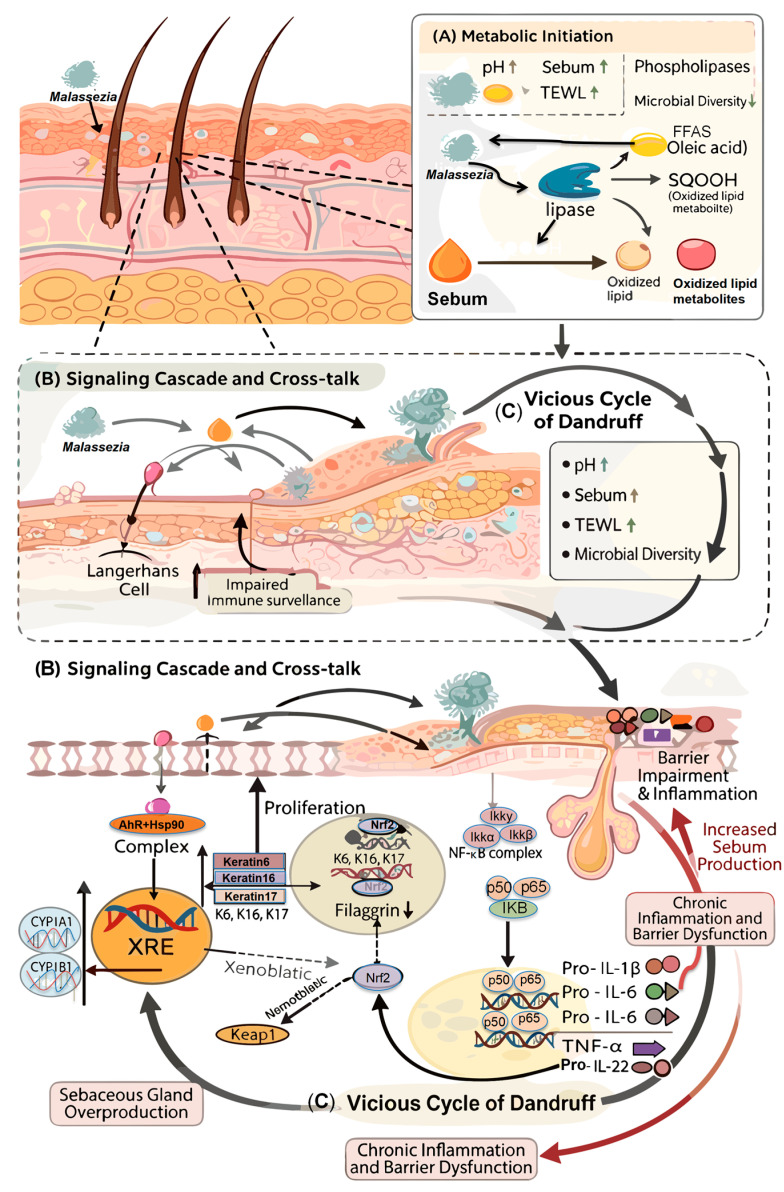
Molecular Mechanisms of *Malassezia*-Host Interaction. This schematic integrates the three-stage pathogenesis: (**A**) Metabolic Initiation: *Malassezia* lipases hydrolyze sebum into FFAs (e.g., oleic acid), while concurrent fungal metabolic oxidation drives squalene peroxidation into SQOOH, and tryptophan metabolism generates indole ligands. (**B**) Signaling Cascade: FFAs and SQOOH trigger NF-κB, leading to transcription of pro-IL-1β and release of pro-inflammatory cytokines (IL-6, TNF-α). Simultaneously, indoles activate AhR, which cross-talks with the Keap1-Nrf2 axis to dysregulate keratin (K6, K16, K17) and filaggrin expression. (**C**) Vicious Cycle: Barrier impairment and inflammation stimulate sebaceous glands to secrete more sebum, providing a continuous nutrient supply for *Malassezia* and sustaining the chronic disease state.Solid black arrows denote metabolic pathways, enzymatic hydrolysis, or molecular translocations; grey intersecting arrows indicate reciprocal biological cross-talk and host-microbe interactions; dashed arrows represent indirect or multi-step regulatory signaling; and bold red curved arrows signify the self-perpetuating vicious cycle.

The interaction between *Malassezia* and host keratinocytes involves a complex network of lipid metabolism and signaling activation (Figure 2).

#### 3.2.1. Lipid Metabolism-Driven Microenvironment Disruption

The lipid-dependent nature of *Malassezia* (with the exception of *M. furfur*) is a primary driver of scalp dyshomeostasis. Lacking genes for de novo fatty-acid synthesis, *Malassezia* species rely on a vast repertoire of lipolytic enzymes—including a multi-gene family of at least 12 secreted lipases and 9 phospholipases across dominant individual species—to hydrolyze host sebum into essential nutrients [4,71].

The resulting free fatty acids (FFAs), particularly oleic acid, penetrate the stratum corneum and perturb keratinocyte biology. These lipids induce hyperproliferation and alter programmed cell death, directly contributing to abnormal desquamation and barrier dysfunction [67,70].

Beyond FFA production, *Malassezia*’s metabolic activity under hypoxic or microaerophilic conditions generates reactive intermediates such as NADH, FADH_2_, and hydrogen peroxide [72]. These oxidative processes lead to the formation of squalene peroxide (SQOOH), a potent oxidative lipid derivative found at significantly higher levels in dandruff-affected scalps. SQOOH further exacerbates oxidative damage to the epidermal barrier [73,74].

Notably, lipid metabolic profiles vary by species: *M. globosa* is characterized by high lipase activity, while *M. restricta* more efficiently catalyzes squalene peroxidation [75,76].

These species-specific differences dictate the varying degrees of microenvironmental alteration and individual susceptibility to scalp disorders. Collectively, this lipase-mediated lipid degradation and the associated metabolic byproducts alter scalp lipid composition and barrier homeostasis. These processes can stimulate sebaceous gland activity and alter sebum composition, thereby forming a pathogenic feedback loop that drives microbial persistence and contributes to the development of *Malassezia*-associated scalp disorders [74]. The major lipid metabolic characteristics of representative *Malassezia* species are summarized in Table 2.

Abbreviations (full terms):

- TAG: Triacylglycerols
- DG: Diacylglycerols
- PC: Phosphatidylcholine
- DGTS: Diacylglyceryltrimethylhomoserine
- FAHFA: Fatty acid esters of hydroxy fatty acids
- FA: Fatty acids
- Cer: Ceramides

#### 3.2.2. Roles of *Malassezia*: From Metabolic Disruption to Immune Activation

*Malassezia* metabolites function as potent signaling ligands that bridge microbial metabolism and host inflammatory responses. This interaction operates through two primary pathways:

Pattern Recognition and Pro-inflammatory Cascade: The cell wall of *Malassezia* is rich in mannan, glucan, and chitin, which serve as primary Pathogen-Associated Molecular Patterns (PAMPs). These are recognized by host receptors such as Dectin-1/2 and Toll-like receptors (TLR2/4). Furthermore, enzymatic activity involving secreted lipases and phospholipases generates free fatty acids (FFAs) and oxidative metabolites (e.g., SQOOH) that activate the NF-κB signaling pathway in keratinocytes. This leads to the robust production of pro-inflammatory cytokines (such as IL-6, IL-8, and TNF-α) and the upregulation of pro-IL-1β transcription, providing the necessary priming signal for subsequent inflammasome activation [8,73,81].

AhR-Mediated Immune Modulation and MalaExs: Indole derivatives from *Malassezia* tryptophan metabolism (e.g., malassezin) serve as ligands for the aryl hydrocarbon receptor (AhR) [81]. Recent evidence also highlights the role of *Malassezia*-derived extracellular vesicles (MalaExs), which carry functional mRNAs and microRNAs (miRNAs). These MalaExs can interfere with host RNA mechanisms, potentially silencing immune-related genes and inducing inflammatory responses [82,83].

Impact on Epidermal Homeostasis: AhR activation dysregulates the expression of differentiation markers, specifically keratins K6, K16, and K17, while down-regulating filaggrin. This molecular shift, often accompanied by the secretion of fungal proteases that degrade extracellular matrix proteins like collagen and elastin, directly contributes to the impaired stratum corneum cohesion and barrier dysfunction characteristic of the dandruff scalp [69,81,84]. Mechanistically, microbial-driven barrier dysfunction acts as the primary gatekeeper and obligate amplifier that translates transient microbial shifts into chronic low-grade (subclinical) inflammation. Quantitatively, microbial-derived lipases and proteases degrade the tight junctions and lamellar lipid integrity of the stratum corneum [4,67]. This physical disruption compromises the scalp’s immunological privilege, permitting an uncontrolled influx of environmental xenobiotics, pollutants, and pathogen-associated molecular patterns (PAMPs) into deeper epidermal layers [41,85,86]. Simultaneously, the physical shearing of keratinocytes triggers the release of endogenous alarmins (such as il-1α or IL-36). This continuous influx locks the host into a self-sustaining loop of sustained immune cell recruitment, meaning that barrier failure directly mediates and sustains the chronicity of the inflammation [86,87]".

Oxidative Stress and Mitochondrial Dysfunction Trigger: Beyond direct receptor binding, *Malassezia* metabolites and associated lipid peroxidation products (such as SQOOH) act as potent instigators of host intracellular oxidative stress [73,88]. The internalization of these irritating ligands by keratinocytes disrupts cellular redox homeostasis, triggering an excessive accumulation of reactive oxygen species (ROS) [73,88]. This sustained oxidative burst directly targets host mitochondria, leading to mitochondrial membrane potential depolarization and structural damage—a state of pronounced mitochondrial dysfunction [88,89]. Consequently, compromised mitochondria leak endogenous danger-associated molecular patterns (DAMPs), specifically mitochondrial DNA (mtDNA) and ROS, into the cytoplasm [89,90]. This intracellular leakage acts as a secondary, synergistic amplification signal that potentiates NF-κB nuclear translocation and lowers the threshold for NLRP3 inflammasome assembly, creating a molecular priming state, thereby laying the groundwork for full-scale host inflammatory escalation under high fungal loads or host susceptibility [89,90,91].

#### 3.2.3. *Malassezia*-Triggered Vicious Cycle

The pathogenesis of dandruff is a self-reinforcing vicious cycle involving lipid metabolism, immune evasion, and barrier failure:

Metabolic Initiation and Adhesion: Surface adhesins (e.g., mannan-binding proteins) facilitate initial attachment, followed by the enzymatic degradation of sebum. This generates irritating FFAs, which directly compromise corneocyte lipid envelopes and accelerate transepidermal water loss (TEWL) [8,70,74].

Barrier-Immune Feedback and Evasion: Structural defects allow deeper metabolite penetration. Simultaneously, *Malassezia* employs immune evasion mechanisms: thick mannan layers can mask β-glucans from detection, while secreted phospholipases interfere with host signal transduction. Persistent AhR activation may further impair immune surveillance by modulating Langerhans cells [69,81,92].

Pathological Reinforcement: The resulting inflammatory milieu and barrier breach stimulate sebaceous glands to increase sebum secretion, providing a continuous nutrient supply for *Malassezia* persistence [5,88,93].

#### 3.2.4. Pathogenic Plasticity and Niche Adaptation: The Dual Nature of *Malassezia*

The pathogenic role of *Malassezia* extends far beyond dandruff, manifesting as a central player in a spectrum of dermatological conditions, including seborrheic dermatitis (SD), atopic dermatitis (AD), *Malassezia* folliculitis, and psoriasis [85,94,95]. The transition of *Malassezia* from a commensal “gatekeeper” to a pathological driver is characterized by its remarkable niche adaptation and pathogenic plasticity.

When the skin barrier is compromised, *Malassezia* does not merely persist; it actively remodels the cutaneous microenvironment to favor its survival. Evidence suggests that in barrier-disrupted skin (such as in AD models), *Malassezia* gains enhanced fitness by up-regulating key metabolic genes involved in nutrient assimilation [86]. This lipid-dependent yeast adapts its metabolic profile to more efficiently utilize the altered lipid landscape of damaged skin, creating a self-sustaining cycle of colonization. By down-regulating essential barrier molecules and ceramides, it further weakens the host defense while simultaneously promoting sebum secretion to ensure a continuous nutrient supply [89,96].

Crucially, the pathogenicity of *Malassezia* is not a uniform trait across the genus but is highly dependent on strain-level diversity. Recent comparative studies reveal that different species, and even different strains within the same species (e.g., *M. restricta* vs. *M. globosa*), exhibit markedly different virulence profiles and evoke distinct molecular responses from the host [86].

Differential Immune Activation: While both *M. restricta* and *M. globosa* can activate the NLRP3 inflammasome, the magnitude of cytokine production (such as IL-1β) in myeloid cells can vary significantly between species [85]. Crucially, while these species possess the inherent capacity for high-magnitude inflammatory activation, this signaling exhibits only a basal, localized priming during dandruff (DF). This low-grade priming is successfully managed by host homeostatic tolerance, and becomes fully unleashed into a destructive cascade only within the hyper-activated, disrupted microenvironments characteristic of overt clinical diseases like SD or AD [5,85].

Genomic Plasticity: Such variations are often attributed to differences in their genomic architecture, specifically in the repertoire of secreted enzymes like lipases and proteases, which dictates their ability to breach the stratum corneum and interact with deeper immune sentinels.

The interaction between *Malassezia* and the host immune system is a double-edged sword:

Pro-inflammatory Provocation: In diseased states, *Malassezia* triggers robust inflammatory responses. It activates the NLRP3 inflammasome via Dectin-2/CARD9 signaling, a process requiring SYK signaling and potassium efflux [85]. Additionally, it drives IL-17-dependent inflammation through the IL-36 receptor/MyD88 axis, which can exacerbate Th2-mediated diseases and potentially reduce the efficacy of targeted therapies like dupilumab [94,96]. Transcriptomic analysis confirms that even in intact skin, *Malassezia* colonization up-regulates genes associated with C-type lectin receptors and neutrophil extracellular trap (NET) formation, although these responses are significantly amplified upon barrier injury [89].

Commensal Protection and Competition: Paradoxically, in a healthy homeostatic state, *Malassezia* serves as a biological shield. It can physically interfere with the invasion of *Staphylococcus aureus* by competing for binding sites like fibronectin on keratinocytes, potentially limiting bacterial virulence through physical contact and heat-labile surface proteins [95].

In conclusion, *Malassezia* functions as a sentinel at the skin barrier. As long as the skin-immune-microbiome axis remains intact, it exists in quiet commensalism. However, once the barrier is breached or immune surveillance shifts, *Malassezia*—exhibiting highly strain-dependent virulence—transitions into an aggressive colonizer. It employs a “scorched earth” strategy: attacking the remaining barrier integrity and down-regulating host defense molecules, while up-regulating its own metabolic machinery to thrive in the dysbiotic niche it helped create.

### 3.3. Divergent Pathological Trajectories: Comparative Spectrum of Dandruff and Seborrheic Dermatitis

Although dandruff (DF) and seborrheic dermatitis (SD) exist on opposite ends of a continuous clinical and pathological spectrum [2,37,97], their differences can be distinctly demarcated by specific thresholds in molecular signaling, microbial community architecture, epidermal barrier status, and host immunological susceptibility [46,87,88]. The transition from the mild, non-inflammatory scaling of dandruff to the severe, erythematous lesions characteristic of SD represents a fundamental, multi-layered breakdown of host-microbe homeostasis [36]. Rather than isolated phenomena, these pathological deviations are driven by an interconnected cascade spanning upstream substrate alterations to downstream genetic and immunological hyper-activation.

#### 3.3.1. Sebum Homeostasis and pH Microenvironment Neutralization

The initiation of both conditions is rooted in the lipid-rich microenvironment of the scalp, yet the quantitative and qualitative kinetics of sebaceous activity dictate divergent clinical paths. Sebaceous glands maintain baseline scalp homeostasis by secreting a complex mixture of triglycerides, wax esters, and squalene [5], forming a hydrophobic film that limits transepidermal water loss (TEWL). Under pathological conditions, however, sebaceous hypersecretion provides an expansive substrate for *Malassezia*’s extensive lipolytic repertoire [8,72]. which in turn releases metabolites that potentially sustain or exacerbate glandular hyperactivity, reinforcing the loop.

In both states, *Malassezia*-derived lipases hydrolyze sebaceous triglycerides, releasing irritating free fatty acids (FFAs), primarily oleic acid [5,98]. In typical dandruff, this process remains localized, driving lower-grade epidermal irritation restricted to superficial desquamation [99]. In the SD microenvironment, however, excess sebum undergoes significant biochemical transformation and oxidative processing, leading to a substantial accumulation of unsaturated FFAs and the formation of squalene peroxide (SQOOH). These lipid derivatives function as potent, aggressive molecular ligands that penetrate the stratum corneum, serving as primary triggers that bridge microbial metabolism with host tissue inflammation [73,74,88].

This lipid dyshomeostasis is further accelerated by a critical “molecular switch”: the regulation of scalp microenvironment pH. The acidic mantle of a healthy scalp (typically pH 4.5–6.0) serves as a fundamental chemical barrier against pathogenic colonization. In DF-affected scalps, this pH balance experiences a moderate shift toward alkalinity [53,100]. Experimental evidence indicates that the lipase and protease activity of *Malassezia* species is highly pH-dependent, with elevated pH levels significantly upregulating the production of irritating metabolites and fungal allergens [101,102]. In SD, this alkaline shift is more pronounced, fundamentally disrupting the structural organization of the host’s lamellar lipid matrix and activating endogenous serine proteases (e.g., Kallikreins). These enzymes accelerate the degradation of corneodesmosomes, facilitating deeper fungal invasion and lowering the threshold for downstream hypersensitivity responses [102].

#### 3.3.2. Epidermal Barrier Macromolecular Integrity and Genetic Susceptibility

The integrity of the stratum corneum barrier largely depends on a well-ordered lamellar lipid matrix (comprising 50% ceramides, 25% cholesterol, and 10–20% FFAs) and robust macromolecular structural frameworks. The divergence between DF and SD can be precisely mapped to the severity of barrier failure and underlying genetic predispositions.

In dandruff, barrier dysfunction remains moderate and structurally confined, presenting clinically as dry, fine white-to-grayish flakes (scales) without macroscopic tissue edema [103,104]. At the biomolecular level, this functional impairment is characterized by loose corneocyte cohesion and a compensatory upregulation of aryl hydrocarbon receptor (AhR)-mediated and hyperproliferation-associated keratins, specifically K16 and K17 [2,105,106]. In susceptible individuals, minor genetic polymorphisms in genes encoding essential envelope proteins—such as filaggrin (FLG)—or innate sensors predispose the scalp to higher *Malassezia* colonization and altered antimicrobial peptide (AMP) expression (e.g., reduced β-defensins) [69,107,108], yet the core epidermal architecture remains intact.

Conversely, the microenvironment of SD experiences a systemic, structural collapse of the epidermal architecture [109]. The baseline expression of critical structural differentiation biomarkers—specifically keratins 1, 10, and 11—is drastically interrupted and downregulated [19,97]. This differentiation arrest is accompanied by a profound depletion of essential intercellular lipids, including ceramides and sphingoid bases [109]. Clinically, this structural failure manifests as the characteristic development of thick, greasy yellow scales overlying well-defined erythematous patches, frequently accompanied by intense pruritus, high TEWL, and transient hair loss [103,109,110].

#### 3.3.3. Microbial Community Architecture: Discrete Imbalance vs. Multi-Kingdom Collapse

The transition from DF to SD corresponds with an ecological escalation from a localized numerical imbalance to a severe, multi-kingdom dysbiosis driven by tissue inflammation and altered lipid substrates [13,54,111].

In dandruff, microbial alterations tend to remain relatively restricted and localized. The scalp generally maintains its core communal architecture, characterized primarily by a localized numerical shift among indigenous symbionts: a discrete elevation of the *Malassezia restricta*/*Malassezia globosa* ratio and a compensatory expansion of *Staphylococcus* species, which closely mirrors a corresponding, moderate decline in resident *Cutibacterium acnes* [53]. This modest taxonomic shift remains confined to the community level and lacks the aggressive virulence factors or polymicrobial complexity required to ignite clinical tissue inflammation [2,53].

In sharp contrast, the microenvironment of SD undergoes a definitive structural collapse of microbial homeostasis [13,54,111]. While dandruff involves a relatively contained fungal shift, SD frequently exhibits a severe, unchecked expansion of *M. restricta* and *M. globosa* [53,111]. Crucially, as a notable point of divergence, severe or immunocompromised SD contexts can lose their historical fungal filtration capacity, allowing the opportunistic invasion and widespread proliferation of non-commensal, external fungi—such as *Candida* and *Aspergillus* species—which are entirely absent in typical dandruff [112,113].

Simultaneously, the bacterial symbiotic balance fundamentally disintegrates in SD. The healthy resident *C. acnes* population undergoes a precipitous, near-total drop compared to the more moderate decrease seen in dandruff [53]. Concurrently, the highly inflammatory tissue exudate and oxidized lipid landscape of SD creates a highly permissive, pathogenic niche. This frequently results in a substantial enrichment and pathological predominance of aggressive invaders—specifically *Staphylococcus aureus*, *Streptococcus* species, and *Acinetobacter*—on lesional skin, converting a stable scalp ecosystem into a dense, polymicrobial entanglement [13,54]. Ultimately, while dandruff represents a largely manageable, localized microbial variation, SD marks an unconstrained, multi-species pathogenic takeover that actively drives chronic cutaneous inflammation and undermines the efficacy of standard mono-targeted antifungal interventions [112,114].

#### 3.3.4. Host Immunological Engagement: Homeostatic Tolerance vs. Cascade Amplification

The definitive boundary distinguishing dandruff from SD lies in the mode of host immunological engagement and underlying endogenous vulnerability, determining whether the host maintains a quiet commensal relationship or succumbs to chronic tissue inflammation.

Dandruff represents a subclinical, homeostatic state where the host immune system maintains a balanced recognition of *Malassezia*, utilizing IgG and IgM for complement-mediated clearance without triggering overt inflammation [9,92,103]. It remains restricted to flake shedding without broad cytokine upregulation, visible erythema, or systemic T-cell infiltration [2,103,109].

In DF-susceptible individuals, minor immune dysregulations can shift this balance toward IgE-mediated hypersensitivity and Th2-polarized responses (elevated IL-4 and IL-13) under an excessive fungal load [1,115], manifesting as itching or transient redness, but the downstream inflammatory cascades typically remain unamplified [99,116].

In SD, this homeostatic tolerance completely breaks down, launching a full-scale cascade amplification. The deep penetration of unsaturated FFAs (oleic acid) and oxidized lipids strongly activates the host’s innate immune system via multiple pattern recognition receptors (PRRs), including Toll-like receptor 2 (TLR-2), NOD-like receptors (NLRs), and C-type lectin receptors [5,87,117]. This initiates the intracellular assembly of the NLRP3 inflammasome, which utilizes the protease caspase-1 to cleave pro-IL-1β into its active, highly potent form [90,100,118]. Concurrently, TLR-2 stimulation drives robust NF-κB activation and subsequent IL-8 production by keratinocytes, amplifying neutrophil and lymphocyte migration into an intense inflammatory amplification loop. In contrast to the basal, low-grade NF-κB activation observed in typical dandruff, the microenvironment of SD exhibits a profound cascade amplification of these downstream inflammatory pathways [116].

Consequently, SD triggers a vast profile of inflammatory markers, including IL-1α, IL-1β, IL-2, IL-4, IL-6, IL-8, IL-10, IL-12, TNF-α, β-defensins, IFN-γ and histamine [109]. Crucially, elevated levels of IL-17-producing -γδT cells are observed, implicating the Th17 axis alongside Th2-driven IL-4 upregulation in its active pathogenesis [119,120].

This intense, full-fledged inflammatory dermatosis is heavily contingent upon endogenous host susceptibilities [5]. In genetically predisposed individuals, specific mutations in genes such as ACT1, C5, IKBKG, STK4, and ZNF750 impair the host’s capacity to restrict *Malassezia* or regulate epithelial differentiation [97,121,122,123]. This genetic vulnerability, coupled with systemic immune dysregulation (e.g., CD4 lymphopenia in HIV contexts [124]) or neurological autonomic dysfunction (e.g., parasympathetic hyperactivity and increased sebum production in Parkinson’s disease [125]), drives the aggressive progression from subclinical scaling into chronic, clinically overt cutaneous tissue inflammation.The comprehensive pathophysiological and molecular differentiations between seborrheic dermatitis and dandruff are summarized in Table 3.

## 4. Integrative Omics and Emerging Molecular Therapeutics

Recent advances in dandruff (DF) research have moved beyond taxonomic surveys toward high-resolution functional mapping. By integrating metagenomics, lipidomics, and transcriptomics, a systemic understanding of the molecular flux at the host–microbe interface has emerged, redefining DF as a state of complex functional dysbiosis.The core research areas, key findings, and methodologies representing these recent advances are systematically summarized in Table 4.

### 4.1. Molecular Insights from Multi-Omics and Lipidomic Profiling

This multi-omics integration has elucidated the biochemical underpinnings of scalp barrier failure and microbial pathogenicity.

#### 4.1.1. Lipidomic Remodeling and Barrier Dysfunction

High-resolution lipidomic profiling has identified specific molecular signatures associated with the compromised DF barrier. Research indicates a significant depletion of long-chain ceramides (classes NP, NH, and AP), which are essential for maintaining the lamellar structure of the stratum corneum [73,74,77]. Conversely, there is a pathological accumulation of unsaturated free fatty acids (FFAs) and oxidative metabolites, notably squalene peroxide (SQOOH). These molecules function as endogenous irritants, increasing transepidermal water loss (TEWL) and activating pro-inflammatory signaling via the NF-κB and MAPK pathways [88,126].

#### 4.1.2. Strain-Level Functional Diversity

Metagenomic evidence highlights that pathogenicity is fundamentally strain-specific. Comparative genomics of *M. restricta* and *M. globosa* has revealed significant intra-species variations in the copy numbers of genes encoding secreted lipases (LIP) and aspartyl proteases (PEP) [67,75]. These enzymes drive the hydrolysis of host triglycerides into irritating FFAs. This functional heterogeneity explains why individuals with similar fungal loads can exhibit divergent clinical phenotypes, as specific virulent strains drive the molecular cascade of inflammation more aggressively [75,102]. Through high-resolution cross-omics correlation, distinct diagnostic and prognostic biomarkers have been identified. At the metabolic level, a down-regulation of functional pathways for biotin and vitamin B6 biosynthesis serves as an early metabolic signature of a failing ecosystem [14], while high ratios of unsaturated free fatty acids and active microbial protease/elastase genes mark active disease progression [127]. Concurrently, host transcriptomic readouts indicate that these strain-level disruptions strongly correlate with the up-regulation of the MAPK/AP-1 and NF-κB pathways, translating to macroscopic elevations in clinical dandruff scores and TEWL [69,73,116,128,129].

**Table 4:** Recent advances in dandruff and scalp microecology research.

Research Area	Key Findings	Methods	Implications	Key References
Microbial Dysbiosis	Beyond *Malassezia*, bacterial community restructuring (e.g., Staphylococci/Propionibacteria ratio) drives pathogenesis.	16S rRNA and Shotgun Metagenomics	Validates the “Microbiome Equilibrium” theory.	[7,14,102]
Strain-Level Diversity	Intra-species functional heterogeneity in lipase and protease expression profiles.	Comparative Genomics	Explains individual variation in dandruff severity.	[67,75,102]
Lipid Remodeling	Significant depletion of long-chain ceramides and elevation of pro-inflammatory oxylipins (e.g., SQOOH).	High-Resolution Lipidomics	Identifies specific lipid biomarkers for barrier failure.	[73,74,126]
Biofilm Architecture	*Malassezia* forms resilient, multi-species biofilms, enhancing recalcitrance to treatment.	SEM/Confocal Imaging and Transcriptomics	Explains the chronic, recurrent nature of dandruff/seborrheic dermatitis.	[82,130,131]
Targeted Modulation	Probiotics and postbiotics successfully recalibrate the microbial ecological niche.	Randomized Clinical Trials (RCTs)	Opens avenues for “ecotherapy” beyond antifungals.	[127,132,133]

### 4.2. Emerging Therapeutic Paradigms: From Eradication to Homeostasis

The therapeutic focus is shifting from broad-spectrum microbial eradication to the precision restoration of the scalp ecosystem. To understand this transition, it is essential to first delineate the molecular basis of current anti-dandruff agents, which serve as the pharmacological baseline for modern formulations.

#### 4.2.1. Molecular Pharmacology of Current Commercial Actives

Existing anti-dandruff interventions primarily utilize molecules that disrupt fungal metabolic viability or normalize epidermal desquamation. Their specific molecular targets and modes of action are synthesized in Table 5.

The anti-dandruff molecules summarized in Table 5 have been widely validated in clinical settings; however, their therapeutic paradigm is currently facing certain potential risks and regulatory scrutiny. For a long time, zinc pyrithione (ZPT) has been regarded as the “gold standard” in the anti-dandruff field due to its excellent inhibitory effect against *Malassezia*. Nevertheless, following its reclassification as a Category 1B reproductive toxicant, ZPT was officially banned in the European Union in 2022. This landmark regulatory shift has not only created a technological vacuum but has also exposed the inherent drawbacks of traditional fungicides.

Beyond safety concerns, conventional intervention strategies suffer from three core mechanistic issues. First, existing antifungal agents primarily target microbial viability but fail to alleviate host scalp inflammation. Evidence indicates that even after fungal clearance, the AhR (aryl hydrocarbon receptor)-mediated signaling axis and pro-inflammatory cytokines (e.g., IL-8) often persist. This leaves the scalp in a “primed” state of subclinical inflammation, making it highly susceptible to rapid recurrence.

Second, broad-spectrum antifungals lack taxonomic specificity. By indiscriminately suppressing beneficial commensals such as *Cutibacterium acnes* (*C. acnes*), these agents further disrupt the scalp microbial balance.

Third, most existing active ingredients are optimized solely against planktonic cells and struggle to effectively penetrate the multispecies biofilm matrix where *Malassezia* resides, leading to persistent refractory symptoms and potentially fostering antifungal resistance.

Consequently, the anti-dandruff field is shifting from “indiscriminate sterilization” toward physiology-based and scalp microecology homeostatic regulation. This underscores an urgent need for next-generation active ingredients—such as naturally derived compounds or targeted bacteriostatic and detoxifying agents—that can break the inflammatory cycle while preserving the integrity of the scalp ecosystem.

#### 4.2.2. Biofilm Interference and Ecological Recalibration

Traditional active ingredients have primarily targeted planktonic cells. However, with advancing research, the recalcitrance of *Malassezia* in dandruff has been linked to multispecies biofilms. Within this matrix constructed by extracellular polymeric substances (EPS), *Cutibacterium acnes* (*C. acnes*) coexists with *Malassezia*. This matrix acts as a physical diffusion barrier, enhancing microbial resistance by 10- to 1000-fold [130,131]. Emerging strategies focus on biofilm disruptors—such as glycoside hydrolases or quorum sensing inhibitors. These agents target the enzymatic pathways of EPS synthesis, thereby improving the penetration and efficacy of conventional actives.

Furthermore, modern microbiome-friendly interventions seek to recalibrate this disrupted microbial niche through targeted ecological modulation. Rather than relying on non-specific antimicrobials, current approaches utilize precise prebiotic and postbiotic matrices to foster the recovery of dominant commensals, thereby re-establishing competitive exclusion dynamics and stimulating host tight junction protein expression [132,133].

Concurrently, topical supplementation with physiological lipid biomimetics—such as pseudo-ceramides—directly addresses downstream barrier vulnerability by neutralizing the localized cascade triggered by oxidative metabolites like squalene peroxide (SQOOH) [127].

#### 4.2.3. Critical Demarcation of Microbiome-Targeted Interventions: Probiotics, Postbiotics, and Transplantation

While ecological recalibration via biotics offers a promising alternative to traditional broad-spectrum antimicrobials, transitioning these concepts into clinical scalp therapeutics requires a rigorous evaluation of their mechanistic boundaries, technological bottlenecks, and safety profiles [137].

The application of live biotherapeutic products (probiotics) to the scalp faces profound formulation and biological constraints [138]. Structurally, traditional hair care formulations rely on broad-spectrum preservation systems (e.g., phenoxyethanol, parabens) to ensure shelf-life, creating an inherent chemical conflict with maintaining live microbial viability. Biologically, the human scalp exhibits immense microenvironmental heterogeneity across different topographical regions [18,28]. Introducing exogenous live strains—often derived from gut niches (e.g., *Lactobacillus* species)—frequently results in poor ecological integration. These transient strains struggle to compete against well-adapted, resident commensals like *Cutibacterium acnes* and *Staphylococcus epidermidis* [139,140]. Furthermore, in severely compromised scalp barriers (e.g., erosive seborrheic dermatitis flares), introducing high-density live biomass poses non-negligible risks of opportunistic localized infection or erratic immunogenicity [141,142].

To circumvent the viability and safety constraints of live cultures, postbiotics—preparations of inanimate microorganisms and/or their components—have emerged as an industrialized alternative [137]. However, their translation into standardized scalp care is hindered by a lack of biochemical uniformity. The preparation of bacterial lysates utilizes diverse inactivation modalities (e.g., thermal processing, ultrasonication, ultra-high-pressure homogenization), which yield completely disparate molecular profiles [138]. For instance, thermal inactivation can denature vital enzymatic fragments, whereas ultrasonication tends to preserve highly immunogenic intracellular proteins and lipoteichoic acids (LTA). Without universally accepted Quality Markers (Q-Markers) to standardize these complex matrices, different batches experience significant “component drift.” Mechanistically, the current literature frequently oversimplifies postbiotic action by attributing clinical outcomes to single pathways (e.g., individual cytokine suppression). In reality, a postbiotic is a highly complex matrix of short-chain fatty acids, peptidoglycans, and bacterial DNA [138], operating within a mechanistic “black box” that presents substantial hurdles for defining pharmacological targets at the scalp-host interface.

Scalp Microbiome Transplantation (SMT), though theoretically capable of resetting a dysbiotic ecosystem by grafting whole healthy donor consortia, introduces severe ecological and safety challenges. The skin microbiome operates as a personalized ecological fingerprint, strictly governed by host genetics and tissue-resident immune cells [18,143]. Forcing a foreign donor microbiome onto a recipient frequently triggers aggressive host-versus-graft immunological rejection or acute competitive friction, causing the transplanted niche to rapidly collapse [28,144]. Moreover, whole-consortia inherent vector risks, including the inadvertent transmission of cryptic pathogens, antibiotic-resistance plasmids, or highly virulent *Malassezia* strains characterized by hyper-expressed lipase profiles. Such blind transplantation could shift the recipient’s scalp into a state of hyper-inflammation, underscoring the need for synthetic, precisely defined consortia over raw donor transfers.

### 4.3. Future Molecular Perspectives: Rationale for Next-Generation Actives

The future of scalp care lies in addressing pathogenic endotypes through targeted molecular intervention. Next-generation therapies are moving toward:

Antagonizing the AhR/STAT3 axis: Developing selective aryl hydrocarbon receptor (AhR) antagonists to block pro-inflammatory signaling triggered by microbial indole derivatives and squalene peroxide (SQOOH), thereby inhibiting K17-mediated keratinocyte hyperproliferation [81,84].

Specific lipase inhibition: Designing inhibitors that specifically target high-virulence lipases (e.g., *Malassezia restricta* LIP1), while preserving the normal metabolic activity of commensal microorganisms [75,102].

Supplemental repair lipidomics: Utilizing longitudinal study data to design “lipidomic biomimetics” that precisely replenish the deficient long-chain ceramide species (including NP, NH, and AP classes) identified in the scalps of dandruff (DF) patients [69,74,88].

## 5. Summary and Future Prospects

### 5.1. The Transition to Precision Scalp Care

While our understanding of dandruff (DF) has evolved from a superficial fungal infection into a sophisticated tripartite pathogenic cascade [17,47]. substantial gaps remain in establishing definitive causality.

Current multi-omics data primarily reflect cross-sectional associations. It remains to be elucidated whether microbial dysbiosis is the primary initiator of the disease or a secondary consequence of a pre-existing host barrier defect.

Future research must transition from descriptive “abundance mapping” to functional validation. Integrating metagenomics, lipidomics, and host transcriptomics is not merely a technical necessity but a conceptual requirement to define individual “pathogenic endotypes,” allowing for a transition from reactive symptom relief to preemptive, personalized intervention [47,84].

### 5.2. Strategic Frontiers: Beyond Antimicrobial Eradication

The future of scalp health lies in promoting a resilient ecosystem rather than indiscriminate antimicrobial eradication. Key areas for molecular-driven investigation include:

Subclinical Monitoring and the “Early Warning” Paradigm: Longitudinal studies suggest that biochemical markers (e.g., histamine and IL-8) may elevate before visible flaking occurs. However, the specificity of these markers for DF versus other subclinical inflammatory states requires further validation. Establishing a reliable “molecular early-warning system” could theoretically enable preemptive care, yet the threshold for clinical intervention remains a subject of debate [84].

Strain-Level Virulence and Functional Metagenomics: Evidence highlights significant functional variations within *M. restricta* and *M. globosa* strains [75,102]. Future research should prioritize clarifying whether specific virulent “strains” or broader community “functional profiles” drive lipase activity and biofilm formation. This distinction is critical for developing truly isoform-specific enzyme inhibitors without disrupting commensal stability.

Precision Microbiome Reconstruction and Host Modulation: Developing next-generation, targeted interventions—such as standardized postbiotic matrices (e.g., inanimate *Lacticaseibacillus paracasei* blends) or precision botanical actives—to selectively suppress opportunistic phenotypes while actively supplementing the metabolic requirements of beneficial commensals like *Cutibacterium* [133,145].

Lipidomic-Driven Barrier Repair: Integrating host lipidomic profiles with microbial ecology is essential for developing biomimetic formulations. However, simply replacing missing lipids may be insufficient if the underlying metabolic “sink”—driven by microbial lipase activity—is not simultaneously addressed [69,126].

### 5.3. Concluding Remarks

In summary, the recurrent issue of dandruff should not be simplistically attributed solely to fungal overgrowth, but rather understood as a complex ecological disorder defined by functional dysbiosis of the scalp microbiome. The synergistic interplay among *Malassezia* metabolic activity, bacterial community restructuring, and host immune signaling—particularly the AhR pathway—collectively establishes a self-reinforcing cycle of “inflammation–barrier impairment” [81,88,131]. Although the potential to elevate dandruff management toward sustained scalp health is substantial, it requires a strategic shift in therapeutic focus: from mere fungal suppression to molecular and ecological rebalancing. By targeting the “residual inflammation” often overlooked by conventional therapies and restoring microecological homeostasis, next-generation treatments hold the promise of definitively breaking the chronic, recurrent cycle that characterizes the course of dandruff.

## Figures and Tables

**Table 1:** The Relationship Between Scalp Microorganisms and Dandruff [13,14,15,17,39,47,53,56,57].

Microorganism	DF/SD Association	Abundance	Direction Notes	References
*M. restricta* (*Malassezia restricta*)	Pathogenic/Promoting	↑ in DF/SD	Correlates with sebum, dandruff score, itch; ↓ scalp hydration	[13,14,15,17,53,56]
*M. globosa* (*Malassezia globosa*)	Pathogenic/Promoting	↑ in DF/SD	Negatively correlated with severity	[14,15,17,53,56]
Unidentified *Malassezia subgroups*	Pathogenic	↑ in DF	Associated with symptom severity	[14,15,17,56]
*Penicillium* spp.	Pathogenic	↑ in DF		[14,15,47,53,56]
*Staphylococcus* spp. (e.g., *S. epidermidis*)	Pathogenic/Promoting	↑ in severe SD	Correlates with TEWL, itch	[13,14,47]
*S. aureus* (*Staphylococcus aureus*)	Pathogenic/Barrier-destructive	↑ in DF/SD		[13,14,15,20,56]
*Streptococcus* spp.	Pathogenic	↑ in DF/SD		[13,14,20]
*C. acnes* (*Cutibacterium acnes*)	Protective	↓ in DF	Dominant in healthy follicles	[14,15,20,47,53,56]
*Pseudomonas* spp.	Protective	↑ in healthy scalp	Pathogenic	[15,47]
*Lactobacillus* spp.	Protective	↓ in DF		[15,17,39]
*Actinobacteria*/*Firmicutes*	Dysbiosis marker	↑ α-diversity in DF/SD	Associated with barrier dysfunction	[13,17,53,56]
*M. restricta*/*C. acnes* ratio	Dysbiosis marker	↑ in DF	Indicates fungal-bacterial imbalance	[14,17,53,56]
*Staphylococcus*/*Cutibacterium* ratio	Dysbiosis marker	↑ in DF	Indicates bacterial imbalance	[14,17,53,56]

Note: ↑ indicates a statistically significant up-regulation or increase; ↓ indicates a statistically significant down-regulation or decrease.

**Table 2:** Lipase Secretion and Lipid Metabolism by the Predominant *Malassezia* Species on the Scalp.

Species	Major Secreted Lipases	Substrate Preference	Optimal Activity Conditions	Dominant Lipid Classes	Key References
*Malassezia restricta*	MrLIP1, MrLIP2, MrLIP3, MrLIP5; additional class 3 and GDSL-like lipases (e.g., MRET_0019, 4032, 1179)	Mainly mono- and diacylglycerols (MrLIP1/2); triglycerides (MrLIP3)	MrLIP1/2 optimal at pH 5, ~34 °C; MrLIP5 active at pH 7–8	Sterols, diacylglycerols (DG), phosphatidylcholine (PC), DGTS	[4,77,78,79]
*Malassezia globosa*	MgLIP1–7; MgMDL2–6	Mono-, di-, and triacylglycerols	pH 5–6; 15–30 °C depending on enzyme	Sterols, DG, PC, DGTS	[77,78,80]
*Malassezia furfur*	MfLIP1 and multiple extracellular lipases/phospholipases	Mono-/di-glycerides; triglycerides; fatty acids	MfLIP1 optimal at pH ~5.8 and ~40 °C	TAG, fatty acids (FA), FAHFA, PE, ceramides	[77,78,80]
*Malassezia sympodialis*	Multiple class 3 lipases and GDSL-like lipases (e.g., MSYG_1326, 2462, 2467)	Predicted mono-, di-, and triacylglycerides	Not well characterized	PC-rich lipid profile with DGTS	[77]

**Table 3:** Pathophysiological and Molecular Differentiations Between Seborrheic Dermatitis and Dandruff.

Comparative Dimension	Seborrheic Dermatitis (SD)	Dandruff (DF)	References
Spectrum Position	Severe, clinical inflammatory pole: Characterized by macroscopically visible erythematous scaly rashes and pronounced tissue inflammation.	Mild, subclinical non-inflammatory pole: Characterized by mild superficial desquamation without prominent visible inflammation.	[2,37,68]
Molecular Signaling	Full-scale cascade amplification: Oleic acid strongly activates PRRs (TLR-2/NLRs) →NLRP3 inflammasome assembly and Caspase-1 activation → robust synthesis of active IL-1β and NF-κB-driven IL-8.	Triglyceride hydrolysis generates free fatty acids, but downstream PRR, NLRP3 inflammasome, and NF-κB cascades remain restricted to baseline, low-grade activation without undergoing full cascade amplification.	[5,91,99,116,117]
Microbial Alteration	Polymicrobial ecosystem dysbiosis: Marked by a notable enrichment and pathological predominance of *Acinetobacter*, *Staphylococcus*, and *Streptococcus* species on lesional skin.	Structural imbalance of resident taxa: Shifts within core indigenous groups; presents as an elevated *M. restricta*/*M. globosa* ratio and an increased *Staphylococcus*/*C. acnes* proportion.	[13,53]
Scalp Barrier Status	Systemic structural failure: Severe downregulation of keratins 1, 10, 11, and profound depletion of ceramides; characterized by high TEWL and epidermal tissue inflammation	Moderate functional impairment: Superficial desquamation with loose corneocyte cohesion; AhR/hyperproliferation-associated keratins (K16, K17) tend to be upregulated.	[2,103,105,106,109]
Immunological Mode	Innate and adaptive hyper-activation: Substantial upregulation of IL-1α/β, IL-6, TNF-α, IL-8, and histamines; can recruit IL-17-expressing γδ T cells; co-dependent on host systemic immune/neural status.	Homeostatic immune tolerance: Maintains subclinical immunological tolerance; restricted to flake shedding without broad cytokine upregulation or systemic T-cell infiltration.	[2,103,109,119]
Clinical Presentation	Greasy yellow scales overlying well-defined erythematous patches; commonly accompanied by intense pruritus, distributing across sebaceous areas.	Dry, fine white-to-grayish scales without visible inflammation; usually restricted exclusively to the scalp.	[103,104,110]

**Table 5:** Molecular targets and mechanisms of action of current anti-dandruff actives.

Active Molecule	Chemical Class	Molecular Target	Mechanism of Action	Ref.
Ketoconazole	Imidazole	Lanosterol 14α-demethylase (CYP51A1)	Inhibits ergosterol biosynthesis; disrupts fungal membrane integrity.	[1,134]
Zinc Pyrithione	Zinc complex	Fe-S cluster proteins/Copper homeostasis	Inhibits mitochondrial respiration via copper-mediated toxicity.	[44,135]
Piroctone Olamine	Pyridone salt	Polyvalent metal ions (Fe^3+^/Al^3+^)	Chelates essential enzymatic cofactors; disrupts energy metabolism.	[44,127]
Selenium Sulfide	Inorganic sulfur	DNA polymerase/Keratinocytes	Cytostatic effect on epidermis; direct fungal cell wall inhibition.	[1,136]
Salicylic Acid	β-Hydroxy acid	Corneodesmosomes	Keratolytic action; facilitates the shedding of hyperkeratotic scales.	[68,136]

## Data Availability

No new data were created or analyzed in this study. Data sharing is not applicable to this article.

## Abbreviations

The following abbreviations are used in this manuscript:

DF	Dandruff
SD	Seborrheic dermatitis
FFA	Free fatty acid
TEWL	Transepidermal water loss
TAG	Triacylglycerols
DG	Diacylglycerols
PC	Phosphatidylcholine
DGTS	Diacylglyceryltrimethylhomoserine
CE	Cholesteryl esters
FAHFA	Fatty acid esters of hydroxy fatty acids
FA	Fatty acids
PE	Phosphatidylethanolamine
Cer	Ceramides
PI	Phosphatidylinositol
